# Identification of specific sequence motif of YopN of *Yersinia pseudotuberculosis* required for systemic infection

**DOI:** 10.1080/21505594.2018.1551709

**Published:** 2018-11-29

**Authors:** Sarp Bamyaci, Roland Nordfelth, Åke Forsberg

**Affiliations:** a Department of Molecular Biology, Umeå Centre for Microbial Research UCMR, Umeå University, Umeå, Sweden; b Department of Molecular Biology, Laboratory for Molecular Infection Medicine MIMS, Umeå University, Umeå, Sweden

**Keywords:** *Yersinia*, T3SS, YopN, mouse infection, virulence

## Abstract

Type III secretion systems (T3SSs) are tightly regulated key virulence mechanisms shared by many Gram-negative pathogens. YopN, one of the substrates, is also crucial in regulation of expression, secretion and activation of the T3SS of pathogenic *Yersinia* species. Interestingly, YopN itself is also targeted into host cells but so far no activity or direct role for YopN inside host cells has been described. Recently, we were able show that the central region of YopN is required for efficient translocation of YopH and YopE into host cells. This was also shown to impact the ability of *Yersinia* to block phagocytosis. One difficulty in studying YopN is to generate mutants that are not impaired in regulation of the T3SS. In this study we extended our previous work and were able to generate specific mutants within the central region of YopN. These mutants were predicted to be crucial for formation of a putative coiled-coil domain (CCD). Similar to the previously described deletion mutant of the central region, these mutants were all impaired in translocation of YopE and YopH. Interestingly, these YopN variants were not translocated into host cells. Importantly, when these mutants were introduced *in cis* on the virulence plasmid, they retained full regulatory function of T3SS expression and secretion. This allowed us to evaluate one of the mutants, *yopN_GAGA_*, in the systemic mouse infection model. Using *in vivo* imaging technology we could verify that the mutant was also attenuated *in vivo* and highly impaired to establish systemic infection.

## Introduction

The type 3 secretion system (T3SS) is an important shared virulence mechanism used by many Gram (-) pathogens to target virulence effectors into host immune cells [1]. The type 3 secretion apparatus (T3SA) is highly conserved both structurally and functionally among the different pathogens [2]. It is composed of a basal body which spans both bacterial membranes to form a base for the needle-filament extending from bacterial surface [2].

Secretion of T3SS substrates has been proven to occur through the T3SA and the needle-filament [3,4]. Most of the characterised T3SS substrates can be divided into two classes, translocators and effectors. The effector substrates are specifically targeted into the host cells by the system and their activity can differ between different pathogens and decide the specific impact of the T3SS on the host defence and infection. For instance while the effectors of the SPI-1 system of *Salmonella* promotes bacterial uptake [5], the effectors of pathogenic *Yersinia* species actively block phagocytosis [6,7]. The translocators on the other hand are essential for targeting of the effector proteins into the host target cells [8]. The exact molecular mechanism for effector translocation has not been demonstrated but so far two different models have been suggested. In the injection model, secretion and translocation are directly connected events. In this model, basal body, needle-filament and translocators form a continuous channel between bacterial and target cell cytosols. As a result, when effector proteins enter the secretion channel, they are translocated in a single step without being exposed to external environment [9]. In contrast, in the two-step mechanism secretion and translocation are not directly connected. Here, in the first step, both translocators and effectors are exported to bacterial surface. By a yet undescribed mechanism a signal is transmitted upon bacteria host cell contact to promote translocator mediated targeting of effectors into the host cells [10,11]. Common in both models is the requirement of a contact between the bacterium and the target cell [1].

In *Yersinia* species, target cell contact can be mimicked *in vitro* by depletion of calcium during growth at 37°C [12,13]. The *in vitro* depletion of calcium is also accompanied by growth cessation [14]. In *Yersinia*, one of the substrates, YopN, has been shown to be involved in regulation and activation of the T3SS while the studies conducted so far have not made it possible to conclude if YopN belongs to the translocator or effector substrates [15]. Under non-inducing conditions or prior to target cell contact, YopN is bound to TyeA [16,17] and this complex is believed to be targeted to the T3SA by YopN chaperones SycN and YscB [18]. The YopN-TyeA complex is hypothesized to repress T3SS expression and secretion via an unknown mechanism from inside the bacteria [16]. It is believed that the secretion of YopN under inducing conditions relieves the repression and results in induction of T3SS expression and secretion [18]. In line with this, in Δ*yopN* mutants the block of T3SS is relieved and substrates are secreted continuously at 37°C. This also results in restricted bacterial replication *in vitro* at 37°C regardless of the calcium concentration [15,18]. Interaction of YopN with inner rod protein, YscI, has been shown to be important in T3SS regulation, suggesting a role for the YopN-YscI interaction in signal transmission [19]. Similarly, in *Shigella* it has been shown that the induction of the T3SS requires secretion of MxiC, which is a homolog of YopN-TyeA expressed as a single protein. Interestingly, the interaction of MxiC with the inner rod MxiI also appears to be involved in regulation [19–21].

Unlike YopN and MxiC, InvE and SepL, two other YopN-TyeA homologues, of *Salmonella* SPI-1 and *E.coli*, respectively, are not secreted [22,23]. Also, another difference is that the Δ*invE* and Δ*sepL* mutants are unable to secrete translocators and are therefore unable to translocate effectors [22–26]. In addition, a Δ*mxiC* mutant of *Shigella* can secrete translocators but secretion is significantly delayed [19]. The results from the studies in *Salmonella, Shigella* and *E. coli* support a secretion hierarchy in these organisms where the translocators are secreted prior to the effectors [20,24,26–28]. In *Yersinia*, however, Δ*yopN* mutants are not impaired for secretion of translocators [29–31]. One difficulty in assigning the role of YopN in virulence is that not only knock-out mutants but also mutants in the genes encoding SycN, YscB and TyeA become deregulated for T3SS expression/secretion and are unable to grow at host temperature, presumably because YopN stability is impaired and levels of YopN become very low [15,29,30,32,33].

Another unique feature of YopN compared to most of its homologs is that it is itself translocated in a T3SS-dependent manner into host cells [34,35]. However, no function has been identified for YopN within target cells, yet. So far the only YopN homolog with a known role within target cells is CopN of *Chlamydia* which functions as a virulence effector [36,37]. However, it should be stressed that most of the other YopN homologues have not been investigated for a role as effector proteins inside host cells.

C-terminal and N-terminal parts of YopN interact with TyeA and the SycN/YscB chaperones respectively and these interactions are, as described above, involved in the regulatory function of YopN [16,17]. We have recently shown that the central region encompassing aa 76–181 is dispensable for the regulatory function of YopN but required for efficient translocation of effectors YopE and YopH in *Yersinia*. In accordance with this, a strain expressing YopN_Δ76–181_-HA failed to block phagocytosis efficiently and was more readily internalized by macrophages [31]. In this study, we identified a putative coiled-coil domain encompassing aa 80–86 within the central region. Mutations predicted to disrupt this domain also had an impact on YopE and YopH translocation and when introduced *in cis* one of the mutants, *yopN_GAGA,_* was also found to be attenuated in the *in vivo* systemic mouse infection model.

## Results

### Construction and characterization of *Y*opN mutants expressed in trans

In a recent study we showed that the central region, encompassing aa residues 76–181 of YopN, with no previously assigned function, was dispensable for the regulatory function of YopN but translocated significantly lower amounts of YopH and YopE into HeLa cells compared to the wt strain. The significance of this finding was also corroborated by the finding that deletion of this region led to impaired blocking of phagocytosis by J774 macrophages [31]. The experiments in the previous study were done by expressing YopN_Δ76–181_-HA from an arabinose inducible promoter *in trans* in a Δ*yopN* mutant background. In order to facilitate *in vivo* animal infection studies we introduced *yopN_Δ76-181_ in cis* on the virulence plasmid of *Y. pseudotuberculosis* strain YPIII. However, when we characterised this strain, we found that it showed a partially deregulated phenotype under non-inducing conditions and intermediate growth phenotype at 37°C, i.e. impaired growth and elevated Yop expression and secretion in non-inducing calcium containing media (data not shown). This phenotype is most likely the result of the lower levels on expression and/or stability of YopN_Δ76-181_. When YopN_Δ76-181_ was expressed under full induction conditions *in trans*, expression levels were high enough to restore regulatory function [31], but when expressed *in cis* from the native promoter, the levels of YopN_Δ76-181_ were apparently too low for restoring regulation. In all, this strain was not suitable for *in vivo* infection studies as an attenuated phenotype in this case could be the result of the slightly impaired growth at 37°C and not necessarily be due to impaired function of YopN in the deletion mutant.

These findings highlight that the regulatory function of YopN to block secretion from the inside of the bacteria is strictly dose dependent and that any study addressing other potential functions of YopN has to verify that intracellular levels of YopN are above the levels required for its regulatory function.

**Figure 1.**
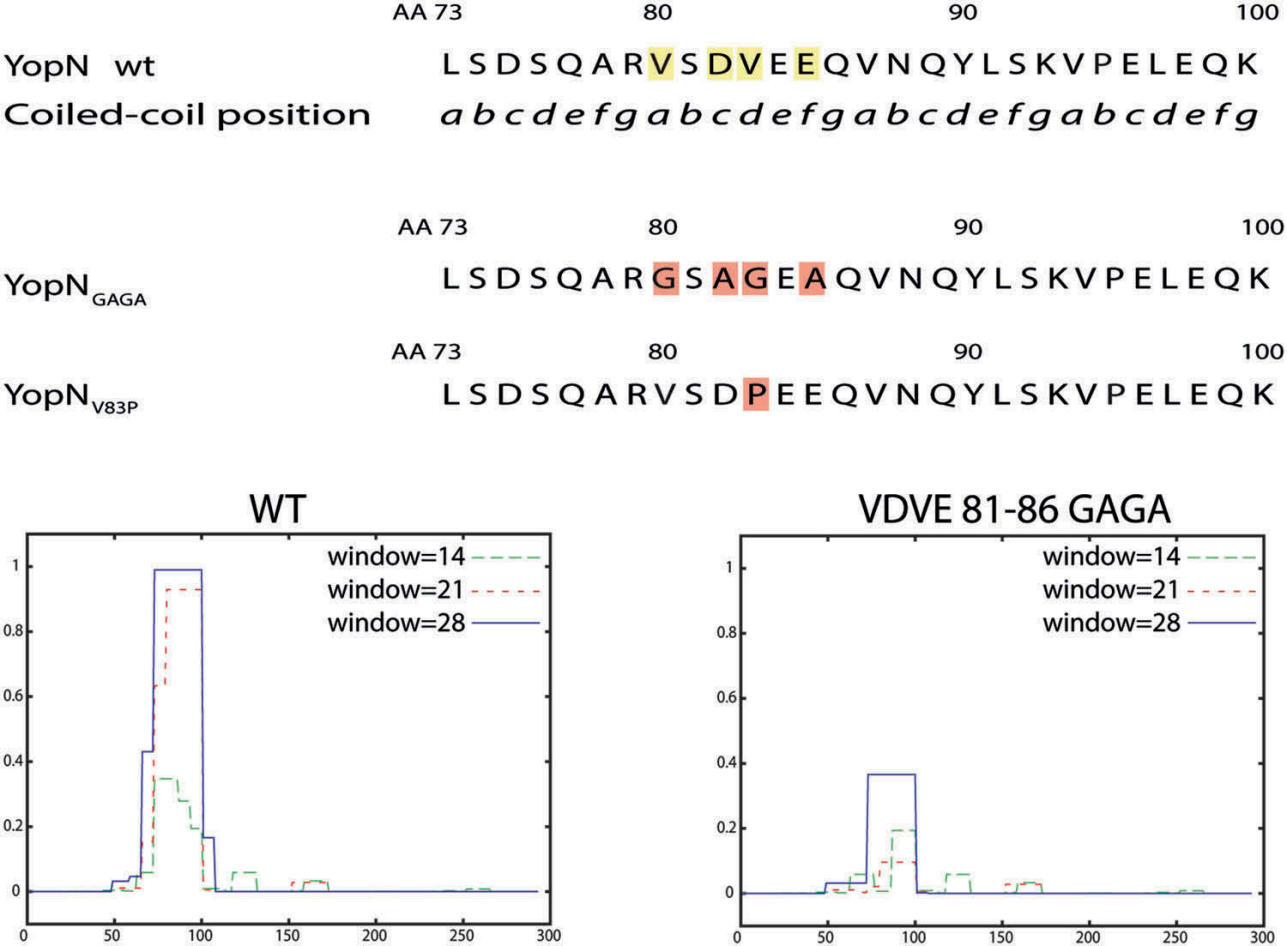
Overview of predicted coiled-coil domain (CCD) and the different constructed mutants. Upper panel shows part of the YopN central region with putative CCM series. Middle panel shows the mutant substitutions constructed, YopN_GAGA_ and YopN_V83P,_ to disrupt the putative CCD. YopN_RARA_ and YopN_SASA_ are similar to YopN_GAGA._, with Arg (YopN_RARA_) and Ser (YopN_SASA_) instead of Gly substitutions. Lower panel shows CCD predictions of wt YopN and YopN_GAGA._

With the aim to facilitate construction of mutants expressed at similar levels as wt YopN we performed an *in silico* analysis of the central region. Thereby we could identify putative coiled-coil domains (CCDs) within amino acids 65–100. A CCD is built up of a series of “abcdefg” coiled-coil motifs (CCMs). A proposed Peptide Velcro hypothesis [38], that outlines elements necessary for the formation of the coiled-coil, states that the “a” and “d” positions of the CCM should be hydrophobic to stabilize the helix dimerization of heterodimeric coiled coils. Between aa 65–100 we could identify three possible CCM repeats (aa 80–100) with hydrophobic residues in the stipulated “a” and “d” positions. From the YopN crystal structure and the predicted three-dimensional structure of YopN [17] amino acids 77 to 90 are likely to form an α-helix including two of the hypothetical CCMs in YopN. Based on this information we decided to make mutations in the first of these CCMs (aa 80–86) within the predicted α-helix (Figure 1). In one approach we changed Val-80 and Val-83 (pos. “a” and “d”) to charged or polar amino acids (Gly, Arg or Ser) and the charged amino acids Asp-82 and Glu-85 (pos. “c” and “e”) to Ala and named the mutants YopN_GAGA_, YopN_RARA_ and YopN_SASA_, to denote Gly, Arg and Ser substitutions respectively. In a second approach we exchanged the position Val-83 (“d”) with a Pro to disrupt the α-helix. This mutation was named YopN_V83P_. We, then, used ExPasy – Coils (https://embnet.vital-it.ch/software/COILS_form.html) [39] to make predictions on the impact of these substitutions on the putative CCD (Figure 1 lower panel and data not shown). All substitutions were predicted to disrupt the putative CCD and each mutated gene was cloned into pBAD24 plasmid under the control of the inducible arabinose promoter. During the cloning procedure, we also added C-terminal hemagglutinin (HA) tags to each mutant to facilitate their detection by Western blot. We have previously shown that a C-terminal HA tag does not interfere with YopN function [31].

**Figure 2.**
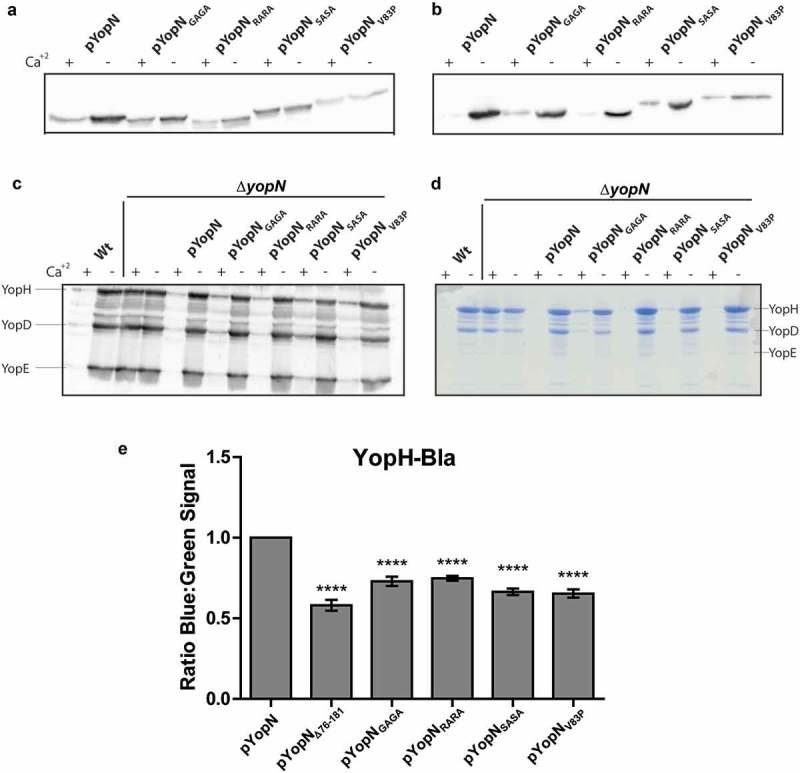
Characterization of the different yopN mutants. *Yersinia* strains expressing the different yopN mutants were grown at 37°C for 3 h in either BHI +Ca^+2^ (non-inducing condition) or BHI – Ca^+2^ (inducing conditions). Whole culture samples (expression) or filtered supernatants precipitated with trichloroacetic acid (TCA) (secretion) were subjected to SDS-PAGE. Expression (a) and secretion (b) of the different YopN variants carrying an HA tag visualized by Western blotting using anti-HA antibody. Total expression of Yops visualized by Western blotting using total-Yop-antisera (c); and secretion of Yops visualized by Coomassie blue staining (d). Analysis of YopH translocation (E). YopH-Bla fusions were introduced instrains expressing the different yopN mutants. Translocation into infected HeLa cells was determined after 30 min by measuring green and blue fluorescence. Translocation levels were calculated as the ratio of blue:green signal after subtracting background signals. The results shown are from 4 independent experiments done as triplicates and normalized to pYopN-HA. The average values ± standard errors of the means (SEM) from 4 independent experiments are shown. All test samples were compared to pYopN using one-way ANOVA followed by the Bonferroni posttest; ****, *P* < 0.0001.

The Δ*yopN* mutant was complemented *in trans* with the different plasmids expressing the YopN variants. The resulting strains were analysed for expression and secretion of the respective proteins. All variants of YopN were found to be expressed and secreted at similar levels as wt YopN in both inducing and non-inducing conditions (Figure 2(a,b)).

Next, we addressed if the YopN variants could complement the deregulated expression and secretion levels of Yop substrates of the Δ*yopN* mutation. All variants complemented both expression and secretion of the substrates to levels comparable to wt YopN (Figure 2(c,d)).

Thus, the strains expressing the different YopN variants predicted to have a disrupted α-helix appeared to function similar to wt YopN in regulation of Yop expression and secretion and the YopN variant itself was also expressed and secreted at levels similar to wt YopN.

### All substitution mutants caused a decrease in YopH translocation

Next, we addressed if the mutations that disrupted the putative CCD motif within the central region of YopN were impaired in Yop effector translocation similar to the recently described central region deletion mutant [31]. The strains described above were introduced *in trans* in a Δ*yopN* mutant background that encoded a YopH_6-99_-Bla (YopH-Bla) fusion protein. The YopH-Bla fusion was then used in the Beta-lactamase reporter assay previously described to measure YopH translocation [31]. Similar to the strain expressing YopN_Δ76-181_-HA, translocation of YopH-Bla into HeLa cells within the first 30 minutes of infection was significantly lower in strains expressing the YopN variants compared to strain expressing the wt YopN (Figure 2(e)). The results verified that the putative CCD within the central region of YopN is important for the efficient translocation of YopH by *Yersinia* T3SS.

### Disruption of the CCD impairs YopN translocation

**Figure 3.**
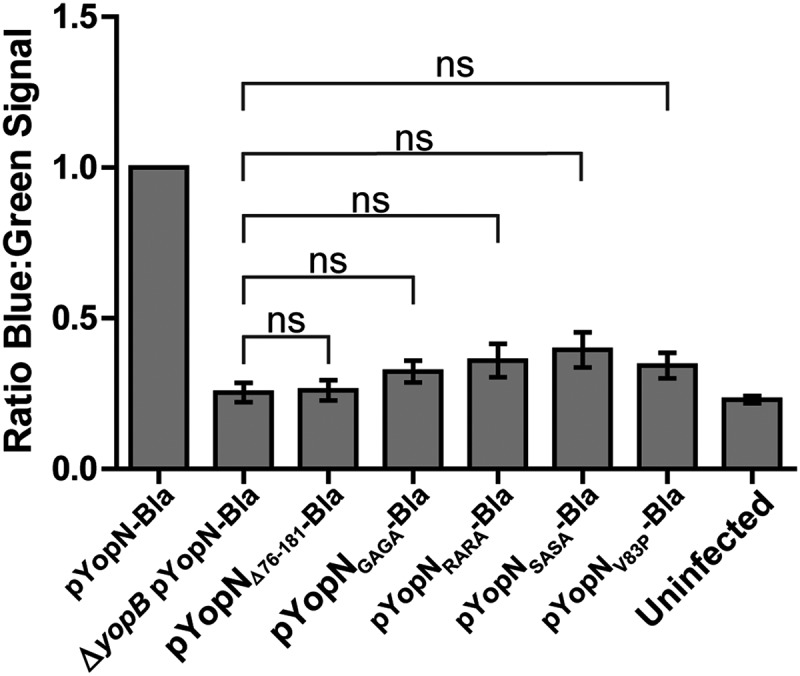
Translocation of the different yopN mutants. Translocation was determined using plasmids expressing C-terminal Bla fusions of the different Yop mutant proteins. These strains were used to infect HeLa cells for 30 min and YopN translocation levels were calculated as the ratio of blue:green fluorescence after subtracting background. The results shown are from 3 independent experiments performed in triplicates and normalized to infection with the strain expressing pYopN-Bla. The mean values ± SEM from 3 independent experiments are shown. All test samples were compared to the Δ*yopB* mutant strain expressing pYopN-Bla using one-way ANOVA followed by the Bonferroni posttest; ns, nonsignificant.

One intriguing difference of YopN compared to homologs from other pathogens is that YopN itself is translocated into target cells by the T3SS [34,35]. InvE and SepL of *Salmonella* SPI-1 and *E. coli* respectively are not secreted to extracellular milieu and are therefore unlikely to be translocated. MxiC of *Shigella* has been shown to be secreted but to our knowledge no one has shown that it is translocated into host cells. The only YopN homolog that has been assigned a function inside host cells is CopN of *Chlamydia* [36,37]. Therefore, we decided to investigate if disruption of the putative CCD had any impact on translocation of YopN itself. To facilitate this we constructed a C-terminal fusion of the full length YopN variants to Bla (YopN-Bla). These constructs as well as constructs encoding YopN_Δ76-181_ and the full-length wt YopN fused to Bla were cloned under the control of the arabinose inducible promoter of pBAD24 plasmid. These plasmids were introduced into the wt strain (YopN^+^) and HeLa cells were infected with strains expressing all the different YopN-Bla variants for 30 minutes. As expected, wt YopN-Bla was found to be translocated, whereas translocation levels of YopN_Δ76-181_-Bla were comparable to the negative control expressing wt YopN-Bla in a Δ*yopB* mutant background. None of the other YopN-Bla variants showed significantly higher translocation levels compared to the Δ*yopB* mutant (Figure 3). From this we can conclude that the putative CCD motif of amino acids 80–86 of YopN is important not only for efficient translocation of YopH but also for translocation of YopN itself.

### Characterisation of yopN_GAGA_ and yopN_RARA_ introduced in cis on the virulence plasmid

**Figure 4.**
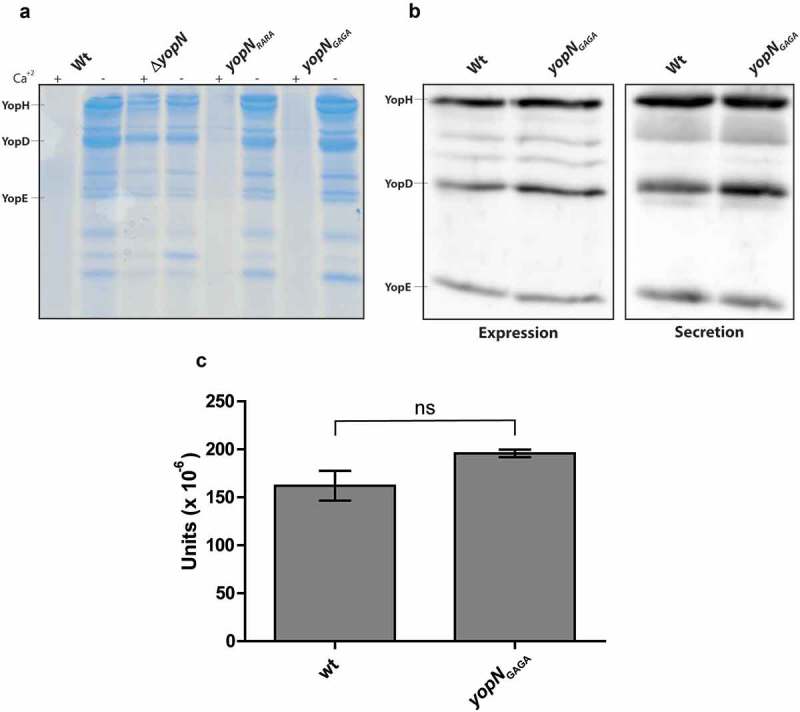
Yop expression and secretion in strains expressing YopN variants *in cis*. (a) The different strains were grown in 37°C for 3 h in either BHI +Ca^+2^ (non-inducing conditions) or BHI – Ca^+2^ (inducing conditions). Filtered supernatants were precipitated with TCA were subjected to SDS-PAGE and secreted proteins were visualized by Coomassie blue staining. (b) The different strains were grown in BHI+Ca^+2^ for 1 h at 37°C before depleting Ca^+2^ to induce the T3SS. After the induction for 30 min, whole cell samples were directly analysed for Yop expression and filtered supernatants were precipitated with TCA and then analysed for Yop secretion by western blotting using total Yop antisera. (c) Secreted YopH levels were quantified by PTPase assay. Strains were grown as in (b) and and PTPase activity in supernatants was analysed as described in materials and methods. 1 unit is equal to 1 mole of product produced per (min x ml supernatant x bacterial OD_660_). The results shown are from 3 independent experiments measured as triplicates. The average values ± SEM from 3 independent experiments are shown. The samples were analysed by Mann-Whitney test, ns, non-significant.

The results from the characterisation of the different mutants within the putative CCD motif of amino acids 80–86 were similar and all had impacts on YopH translocation and on translocation of YopN itself. As the levels of expression of the YopN variants also were similar to the levels of wt YopN, we decided to introduce the *yopN_RARA_* and *yopN_GAGA_* mutations *in cis* on the virulence plasmid by double cross-over recombination. The Yop secretion profiles of the *yopN* mutants and the wt strain were compared after 3 h of growth at 37°C (Figure 4(a)). Whereas the Δ*yopN* mutant, as expected, secreted T3SS substrates both in the presence and absence of Ca^+2^, the secretion levels in the *yopN_RARA_* and *yopN_GAGA_* mutants were comparable to those of the wt strain under both inducing and non-inducing conditions. Since translocation assays are focused on efficient early translocation, we wanted to verify that the *yopN_GAGA_* mutant expressed and secreted similar levels of Yops also at early time points after induction. Total expression and secretion of Yops were therefore analysed 30 minutes after induction. As can be seen in Figure 4(b), both expression and secretion levels were similar when the wt strain and the *yopN_GAGA_* mutant strain were compared. Secretion levels are quite low 30 minutes after induction. Therefore, in order to compare and quantify secretion levels we used the acid phosphatase activity of YopH to quantify secretion of YopH in the wt and *yopN_GAGA_* mutant strains. It has previously been shown that YopH is the only acid phosphatase activity in *Y. pseudotuber-culosis* [40]. We also verified that a Δ*yopH* mutant was completely devoid of phosphatase activity (data not shown). As seen in Figure 4(c), the acid phosphatase activity in supernatants from wt and the *yopN_GAGA_* mutant strains 30 minutes after induction of Yop secretion were similar and the statistical analysis verified that there was no significant difference between the strains. In summary these experiments verify that the *yopN_GAGA_* mutant strain expresses and secretes similar levels of Yop effectors as the wt strain.

### The yopN_GAGA_ cis mutant is impaired for YopH and YopE translocation

**Figure 5.**
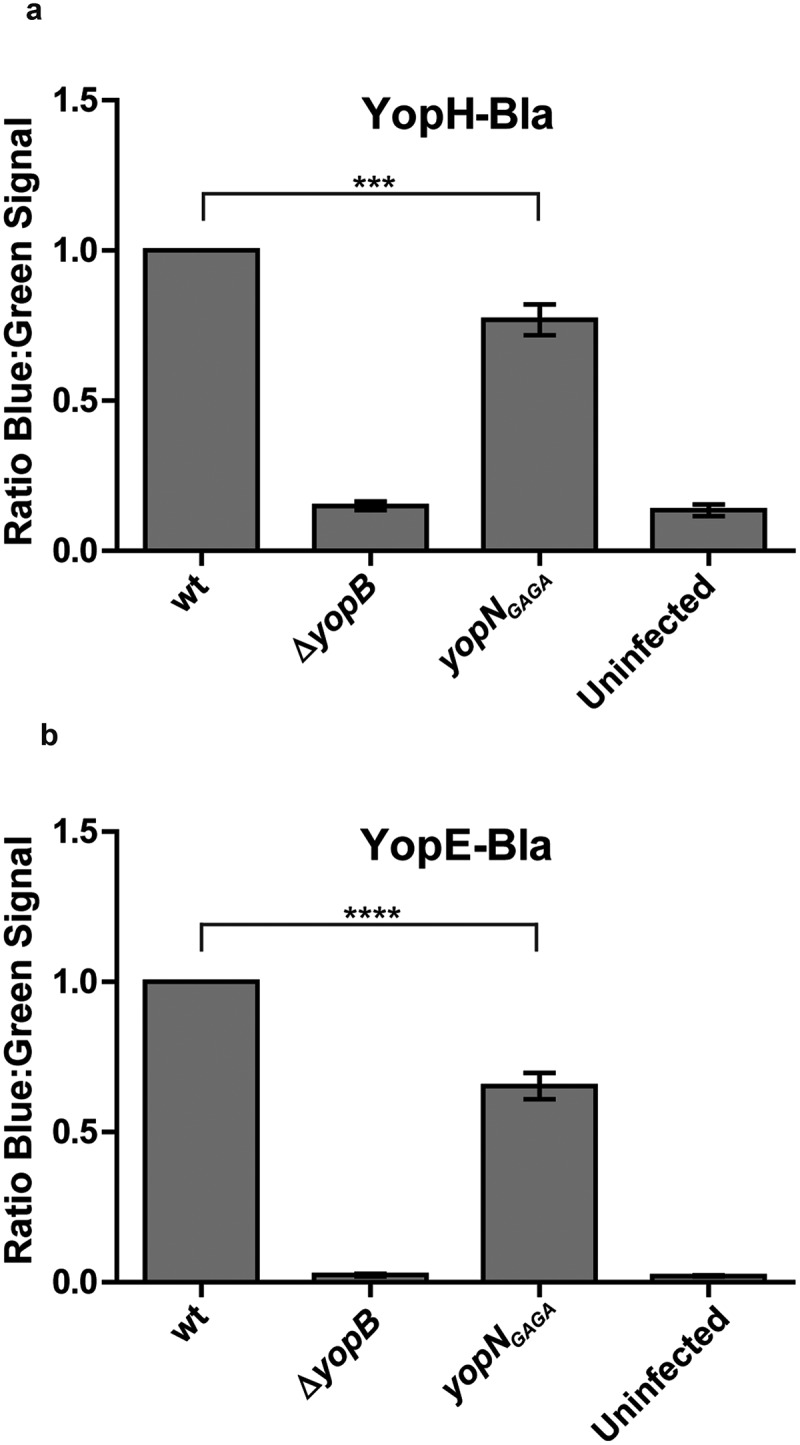
YopH and YopE translocation in strains expressing YopN_GAGA_ *in cis*. YopH-Bla (a) or YopE-Bla (b) were introduced into wt, Δ*yopB* and *yopN_GAGA_* strains. HeLa cells were infected with the different strains for 30 min. Translocation levels were calculated as the ratio of blue:green signals after subtracting background levels. The results shown are from 3 independent experiments done as triplicates and normalised to cells infected with the wt strain. The mean values ± SEM from 3 independent experiments are shown. *yopN_GAGA_* samples were compared to wt using one-way ANOVA followed by the Bonferroni posttest; ***, *P* < 0.001.; ****, *P* < 0.0001.

Next we decided to verify if the mutants, when introduced *in cis*, also were impaired in Yop effector translocation. We therefore introduced YopH-Bla and YopE_6-86_-Bla (YopE-Bla) into the *yopN_GAGA_* mutant *in cis* on the virulence plasmid. As negative controls, we also included strains expressing the YopH-Bla and YopE-Bla fusions in Δ*yopB* mutant background. Using the beta-lactamase reporter assay, we could confirm that both YopH and YopE translocation levels were significantly lower in the mutant compared to the wt strain after 30 minutes of infection (Figure 5). The impact on YopE translocation was somewhat bigger compared to YopH. This is similar to our previous findings for YopN_Δ76-181_ where the reduction in translocation levels were higher for YopE than YopH [31]. In summary, we can conclude that the *yopN_GAGA_* mutation has an impact on the translocation efficiency of both YopH and YopE.

### The yopN_GAGA_ CCD mutant is attenuated in the systemic mouse infection model

Studies of a more direct role for YopN in infection and virulence have been hampered by the fact that the Δ*yopN* mutants characterised are deregulated for Yop expression, secretion and impaired for growth at 37°C [15,18]. The aim of this study was to identify and characterise YopN mutants with retained regulatory function and growth phenotype similar to the wt strain. The *yopN_GAGA_* mutant, where the putative CCD motif was disrupted, fulfilled these criteria. Therefore, we introduced the *yopN_GAGA_* mutation into the Xen4 YPIII/pIB102 strain that expresses luciferase and can be readily monitored in real time during infection by the *In vivo* Imaging System (IVIS, PerkinElmer). Using this technology allowed us to verify if the observed impact on YopE and YopH translocation and translocation of YopN itself also resulted in attenuation of the progress and spread of the infection. Groups of four mice were infected orally with either wt Xen4 or Xen4 *yopN_GAGA_*. According to CFU counts and the volumes of bacterial suspension consumed by mice, on average the infection doses for each mouse was 3.8 × 10^8^ bacteria for the wt strain and 4.4 × 10^8^ bacteria for the *yopN_GAGA_* mutant strain.

**Figure 6.**
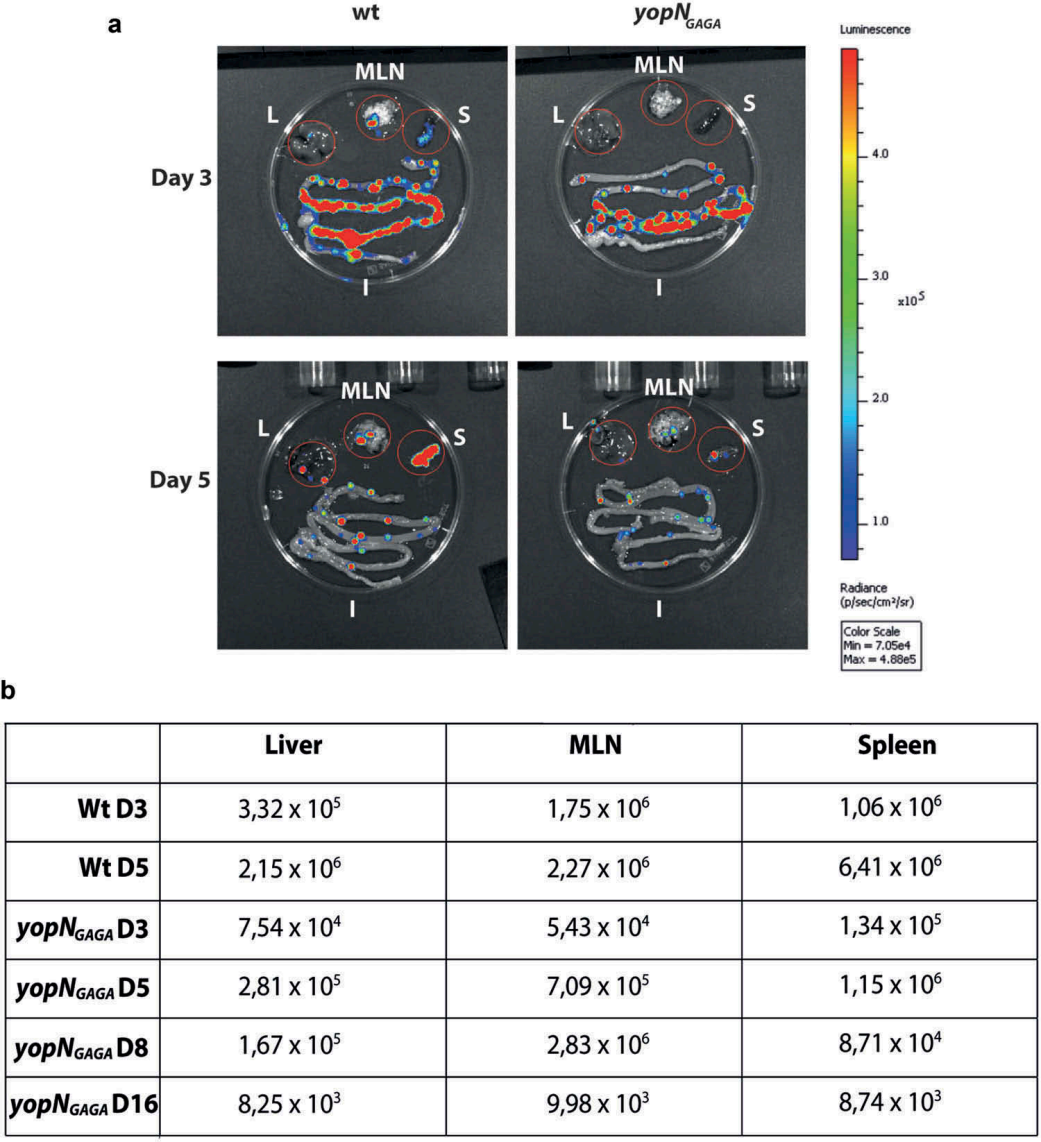
Infection of BALB/c mice with bioluminescent *Yersinia* wt and *yopN_GAGA_* mutant. The infection was monitored using bioluminescent imaging for up to 16 days post infection (p.i.). (a) At each of days 3 and 5 p.i. one mouse infected with wt and one mouse infected with *yopN_GAGA_* were sacrificed. In all these mice, bacterial luminescence was measured from dissected liver (L), mesenteric lymph nodes (MLN), spleen (S) and intestines (I). (b) In addition to mice sacrificed in panel (a), at each of days 8 and 16 p.i. one mouse infected with *yopN_GAGA_* were sacrificed. The table shows luminescence levels from dissected livers, MLNs and spleens at 3 and 5 days p.i for wt and 3, 5, 8 and 16days p.i. for *yopN_GAGA._*

Luciferase was initially monitored for all mice at day 3 and 5 post-infection (p.i.) and each day one mouse infected with the wt and one infected with the *yopN_GAGA_* mutant were sacrificed to monitor the localization of bacteria within intestines, mesenteric lymph nodes (MLNs), spleen and liver. Already by day 3 p.i., both strains had successfully colonised the intestines including Peyer´s patches (Figure 6(a) upper panel). Interestingly, the wt bacteria had also initiated a systemic infection already at day 3 p.i. by spreading into MLNs as well as to spleen and liver, whereas the *yopN_GAGA_* had not spread beyond the intestine (Figure 6(a) upper panel and Figure 6(b)). At day 5 p.i., the sacrificed mice revealed that the wt strain had spread further to establish systemic infection with high signal levels in both spleen and liver. The mutant strain showed increased levels in MLNs but still showed low levels in spleen and liver compared to wt (Figure 6(a,b)). At day 5 p.i., all mice infected with the wt strain showed severe symptoms of infection with low mobility and weight loss while mice infected with the mutant strain did not show any apparent symptoms from the infection. All mice infected by wt were judged to be terminally ill and sacrificed at day 5 p.i.. The signals obtained from the MLNs of a sacrificed mouse infected with the *yopN_GAGA_* strain increased at day 8 p.i. while the signal levels in liver and spleen were lower at day 8 p.i. compared to day 5 p.i.. At day 16 p.i. the remaining mouse infected with the mutant strain had cleared the infection with only weak signals in each organ very similar to background levels (Figure 6(b)). Our infection experiment shows that there are differences in the infection between individual mice but overall our monitoring of the progress of the infection in the two strains revealed differences between the strains that showed that mutant was attenuated in the systemic mouse infection model.

## Discussion

T3SSs are key virulence mechanisms shared by many Gram-negative pathogens that target the host defence to promote infection. They are complex, involve a large number of genes and are tightly regulated to avoid expressing the genes involved when they are not needed. In pathogenic *Yersinia*, YopN has been extensively studied and identified as one of the key regulators of Yop expression and secretion [15]. In addition to its regulatory role, YopN has been shown to be targeted into host cells. Recently, we showed that YopN is also required for efficient translocation of YopE and YopH and, thereby, for blocking phagocytosis. In this work we have generated mutants within a putative CCD domain with fully retained regulatory function and thereby for the first time been able to verify that YopN indeed has a direct role in promoting systemic infection in the *in vivo* mouse infection model for *Yersinia.*

We recently identified a central region encompassing aa 76-181 of YopN as important for efficient early translocation of YopE and YopH during *in vitro* infection of HeLa cells and this also correlated to impaired ability to block phagocytosis by infected J774 cells [31]. Phagocytosis inhibition has previously been shown to be a key T3SS dependent virulence mechanism of *Yersinia*. Early translocation of Yop effectors is essential for efficient blocking of phagocytosis. Previous studies have shown that the tyrosine phosphatase YopH targets the focal adhesion complex and rapidly dephophorylates proteins like focal adhesion kinase (FAK) to interfere with phagocytosis [41–44]. YopH and YopE mutants are also unable to spread beyond MLNs to spleen and liver to cause systemic infection [45,46]. Based on these previous findings, it is likely that our findings that the putative CCD within the central region of YopN is required for efficient early translocation of YopE and YopH directly contributes to the attenuation of the *yopN_GAGA_* mutant in the systemic mouse infection model for *Yersinia.*

In addition to the impact on translocation of YopE and YopH, we also identified another novel property linked to the putative CCD within the central region of YopN. All substitution mutant proteins together with the YopN_Δ76-181_ mutant protein lost the ability to be translocated into host cells. The putative CCD maps to the region just downstream of the domain binding the two chaperones SycN and YscB. In other T3SS effectors, the domain required for translocation has been mapped to similar regions within and/or just downstream of the chaperone-binding domain [47–49]. So far no target protein or phenotype linked to intracellular targeting of YopN has been identified. The only YopN homologue of other T3SSs with an established intracellular role so far is CopN of *Chlamydia* [36,37]. When CopN was expressed in yeast cells, it resulted in growth inhibition and defects in cell division. In these studies YopN was also expressed in yeast cells but no specific phenotype related to expression of YopN in yeast cells was identified. The previous limited studies make it difficult to predict the impact of lack of intracellular targeting of YopN. It is tempting to speculate that YopN could have a significant role inside infected host cells as all other translocated Yop effectors have been found specifically target intracellular host cell proteins [9,50]. Therefore, we have initiated a yeast two-hybrid study in an attempt to identify any intracellular eukaryotic proteins binding to YopN.

In the systemic oral infection model in mice, *Yersinia* reaches the Peyer’s Patches (PPs) within the small intestine [51]. Thereafter the infection spreads to cecum and MLN. From the infected MLNs, the T3SS facilitates the further dissemination into spleen, liver and the bloodstream to cause a systemic infection [52]. Mutations in either of the main effectors YopE and YopH render *Yersinia* unable to spread from the MLNs [45,46]. In this study we used a bioluminescent Xen4 strain to monitor and compare the wt and *yopN_GAGA_* mutant strains in orally infected mice. *In vivo* imaging using bioluminescent bacteria is an excellent method to monitor the progress of infection and to compare different strains using a limited number of mice. The results from *in vivo* infection showed that the *yopN_GAGA_* strain only showed limited systemic spread and symptoms and the infected mice were able to clear the infection. In summary, this shows that the putative CCD domain within the central region of YopN is indeed also required for systemic infection in the mouse infection model.

The fact that YopN is targeted into host cells by the T3SSs is intriguing. However, since the mutants studied so far have been regulatory mutants where Yop expression and secretion is deregulated and with impaired growth at 37°C, it has not been possible to address a more direct role of YopN in promoting *in vivo* infection. The role of YopN as a negative regulator involves interactions with at least three additional proteins. One is TyeA that binds YopN via TyeA binding domain located at the C-terminal of YopN [16,17]. Mutations within YopN or TyeA that disrupt this interaction result in deregulation of expression and secretion and impaired growth at 37°C [53]. The TyeA-YopN complex also binds to the YopN chaperones SycN and YscB via the N-terminal domain of YopN [17]. It is believed that the chaperones are responsible for introducing the YopN-TyeA complex to the basal body of T3SS [18]. These complexes are stable only at non-inducing conditions, i.e. prior to target cell contact or at high Ca^+2^ concentrations at 37°C. This complex blocks T3SS substrate export by a yet unknown mechanism [16]. When the bacteria encounter inducing conditions (after target cell contact or in Ca^+2^ depletion at 37°C), these interactions are destabilized and YopN itself becomes a substrate of the T3SS and is secreted. Export of YopN relieves suppression and not only the expression of the T3SS is upregulated but also secretion is induced [18]. In the absence of either YopN or its binding partners, the inability to form the complex results in constitutive activation of the system at 37°C accompanied by growth restriction [15,29,30,32]. Lack of the chaperones SycN and/or YscB is likely to lower the stability and levels of YopN that in turn could result in a regulatory phenotype. In our recent study we identified a central region of YopN (aa 76-181) that was dispensable for regulation. However, the expression levels of YopN_Δ76-181_ were lower compared to wt YopN in titration experiments where the proteins were expressed from an arabinose inducible promoter. We could verify that the minimum arabinose level required for full regulatory function were almost 7 times lower in wt YopN than for YopN_Δ76-181_ [31]. This could either be due to lower stability or less efficient translation of the mutant. To facilitate *in vivo* studies it is necessary to introduce the *yopN* mutations *in cis* on the virulence plasmid. When we introduced the *yopN*_Δ76-181_ *in cis*, the resulting strain was deregulated for Yop expression and secretion. This, most likely, is a consequence of too low YopN expression levels to promote full regulatory function when expressed from its native promoter. Therefore, we undertook an *in silico* analysis of the central region with the aim to identify a small domain/motif that could be targeted by mutagenesis and where the mutant YopN would have similar stability/expression levels as wt YopN. Within the central region we could identify a putative coiled-coil domain (CCD) between aa 80–86 within an alpha helix. Introduction of amino acids predicted to disrupt the CCD were generated and the resulting YopN variants were verified to be expressed at similar levels as wt YopN and importantly with fully retained regulatory function. Further, the mutants, similar to the central region deletion mutant, were also found to have an impact on Yop translocation when expressed *in trans*. Altogether, this illustrates the measures required to evaluate a more direct role for YopN in the *in vivo* virulence of *Yersinia.*

So far no T3SS proteins or host proteins have been verified to interact with the central region of YopN. Our finding that a putative CCD is required for efficient Yop effector translocation, translocation of YopN as well as *in vivo* virulence suggest that YopN via this identified putative CCD domain is a potential target to interact with several proteins. The impact on Yop effector translocation and on YopN’s own translocation indicates that YopN could interact with proteins involved in translocation, i.e. YopB, YopD, LcrV, YopE and YopK. YopN, is a negative regulator that provides timely export of not only effector proteins but also translocator proteins [15,29,31,53]. Homologs from other pathogens like InvE in *Salmonella* SPI-1, SepL in *E.coli* and MxiC in *Shigella* have somewhat different role in being required for translocator secretion, albeit at different levels [19,23,24,26]. Common to all these homologues is that they are likely to interact with T3SS substrates but with different activities and roles. Amino acids in the putative CCD are required for *in vivo* systemic infection and are likely to be involved in protein-protein interactions presumably both within the central region of YopN as well as with other T3SS proteins involved in translocation. Both hydrophobic translocators YopB and YopD, as well as their homologs in other systems, have been shown to carry CCDs [8]. Moreover, since needle filament, tip protein and hydrophobic translocators carry CCDs, CCDs were suggested to be important for a functional translocator complex [54]. Thus, it would not be surprising if other proteins involved in translocation possess CCDs.

Since YopN itself is translocated, it is possible that key interactions could also occur at the level of the host cell membrane or inside the host cell. Here, it is intriguing to note that all of YopK, YopE and YopD, which are involved in translocation, are also targeted into host cells [55,56]. Importantly, YopK is known the regulate translocation from inside the target cell indicating that translocation can be regulated from there as well [55]. In addition, it is also possible that the central region of YopN interacts with host protein(s) when internalised into host cells by the T3SS.

The YopN homologues InvE, SepL and MxiC from *Salmonella, E. coli* and *Shigella* have been described to be required for timely secretion of the T3SS translocators and therefore have also been described to be part of an intracellular sorting platform [20,24,26–28]. The sorting platform is described as a multi-protein complex to ensure ordered secretion of the T3SS substrates where the translocators are secreted before the effectors [26]. YopN is not required for translocator secretion [29–31] but its function is linked to translocation and most likely by somehow interacting with the translocators influencing their function. Another difference is that InvE and SepL are either not secreted or secreted at low levels [22,23]. This makes it somewhat unlikely that they would, similar to YopN, also be targeted into host cells and act as potential effectors. In InvE, SepL and MxiC, the corresponding TyeA homologues are encoded in the same reading frame constituting the C-terminal part of these proteins. In some other pathogens like *Pseudomonas, tyeA*, similar to *Yersinia*, is a separate gene and it is therefore possible that the *Pseudomonas* homologue PopN could have a similar multifunctional role as YopN and also be targeted into host cells. Interestingly, the central region of PopN also appears to encode a putative CCD (unpublished data). However, so far no studies to address if PopN is also a potential T3SS effector in *Pseudomonas* have been undertaken.

Herein, we have for the first time verified that YopN has a direct role in virulence and is required for systemic infection in the *in vivo* mouse model for *Yersinia*. This role is in addition to the previously identified role in fine-tuning regulation and secretion of Yop effectors and establishes that YopN is a key multifunctional protein with a novel role in promoting efficient translocation of Yop effectors.

## Materials and methods

### Bacterial strains and growth conditions

All bacterial strains and plasmids used in this study are described in Table 1. Unless otherwise stated, bacteria were grown in Luria-Bertani (LB) growth medium or on LB agar. Incubation temperatures were 26°C for *Yersinia* and 37°C for *E.coli*. When needed, antibiotic concentrations were used as following: kanamycin, 30 µg/ml; chloramphenicol, 25 µg/ml; and carbenicillin, 100 µg/ml. Conditions to induce T3SS in *Yersinia* were explained in corresponding section.

**Table 1.** Strains and plasmids used in this study.

Strain or plasmid	Relevant genotype	Reference
**Strains** *Escherichia coli*		
S17-1λ*pir*	RP4-2 Tc::Mu-Km::Tn7 (λ*pir)*	[57]
*Yersinia pseudotuberculosis*		
YPIII pIB102	*yadA*, Km^r^ (wild type)	[12]
YPIII pIB604	pIB102: *yopB*, Km^r^	[59]
YPIII pIB822 YPIII pIB29	pIB102: *yopN*, Km^r^ pIB102: *yopH*, Km^r^	[31] [58]
YPIII pIB823	pIB102 expressing YopN_RARA_, Km^r^	This study
YPIII pIB825	pIB102 expressing YopN_GAGA_, Km^r^	This study
YPIII pIB102 YopH-Bla	pIB102 expressing YopH_6-99_-Bla_24-286_, Km^r^, Cml^r^	[61]
YPIII pIB604 YopH-Bla	pIB604 expressing YopH_6-99_-Bla_24-286_, Km^r^, Cml^r^	This study
YPIII pIB822 YopH-Bla	pIB822 expressing YopH_6-99_-Bla_24-286_, Km^r^, Cml^r^	[31]
YPIII pIB825 YopH-Bla	pIB825 expressing YopH_6-99_-Bla_24-286_, Km^r^, Cml^r^	This study
YPIII pIB102 YopE-Bla	pIB102 expressing YopE_6-86_-Bla_24-286_, Km^r^, Cml^r^	[61]
YPIII pIB604 YopE-Bla	pIB604 expressing YopE_6-86_-Bla_24-286_, Km^r^, Cml^r^	This study
YPIII pIB825 YopE-Bla	pIB825 expressing YopE_6-86_-Bla_24-286_, Km^r^, Cml^r^	This study
Xen4 pIB102	pIB102: *Tn1000::Tn5 luxCDABE*, Km^r^	Caliper Life Science
Xen4 pIB825	Xen4 pIB102 expressing YopN_GAGA_, Km^r^	This study
**Plasmids**		
pSB1	pBAD24 YopN-HA	[31]
pRN62	pBAD24 YopN_Δ76-181_-HA	[31]
pRN65	pBAD24 YopN_RARA_-HA	This study
pRN66	pBAD24 YopN_V83P_-HA	This study
pRN67	pBAD24 YopN_SASA_-HA	This study
pRN68	pBAD24 YopN_GAGA_-HA	This study
pRN69	pBAD24 YopN_RARA_-Bla_24-286_	This study
pRN70	pBAD24 YopN_SASA_-Bla_24-286_	This study
pRN71	pBAD24 YopN_GAGA_-Bla_24-286_	This study
pRN72	pBAD24 YopN_V83P_-Bla_24-286_	This study
pRN73	pDM4 containing *yopN_RARA_* mutation	This study
pRN75	pDM4 containing *yopN_GAGA_* mutation	This study
pSB22	pBAD24 YopN-Bla_24-286_	This study
pSB23	pBAD24 YopN_Δ76-181_-Bla_24-286_	This study
pNQ705-H-Bla	pNQ containing YopH_6-99_-β-lactamase fusion	[61]
pNQ705-E-Bla	pNQ containing YopE_6-86_-β-lactamase fusion	[61]

Cultivation of HeLa cells was done in modified Eagle medium (MEM) supplemented with 10% fetal calf serum (FCS), 2 mM glutamine, 0.035% sodium bicarbonate and 100 IU/ml penicillin. The cells were incubated at 37°C with 5% CO_2_.

### Construction and cloning of different yopN mutants

In order to disrupt the predicted coiled-coil motif (CCD), we focused on amino acids 80–86 of YopN, all predicted to form an alpha helix [17] (accession code 1XKP in PDB). Three different mutants were constructed by changing the charged amino acids Asp-82 and Glu-85 to Ala together with changing Val-80 and Val-83 to polar or charged acids: Arg (pYopN_RARA_; pRN65), Gly (pYopN_GAGA_; pRN68) or Ser (pYopN_SASA_; pRN67). In the second approach we substituted Val-83 into Pro to disrupt the alpha helix (pYopN_V83P_; pRN66). pBAD24-YopN-HA (pSB1) was used as a template to amplify two different fragments and the hemagglutinin (HA) tag was kept at the C-termini of downstream fragments. Both fragments amplified for each mutation were introduced into the pBAD24 vector, digested with EcoRI and HindIII, using In-Fusion HD cloning kit (Clonetech Laboratories, Inc.) according to the manufacturer’s protocol. The mutations were confirmed by sequencing (GATC Biotech AB, Stockholm, Sweden). Isolated plasmids were then electroporated into Δ*yopN* and Δ*yopN yopH-bla*.

To generate C-terminal Bla fusions of the genes encoding the YopN variants above, full length *yopN_RARA_* (pRN69), *yopN_GAGA_* (pRN71), *yopN_SASA_* (pRN70) and *yopN_V83P_* (pRN72) genes were amplified from using the plasmids described above as templates. Amplification of wt *yopN* (pSB22) and *yopN_Δ76-181_* (pSB23) were done from wt *Yersinia pseudotuberculosis* YPIII pIB102 strain and plasmid pBAD24-YopN_Δ76-181_-HA (pRN62), respectively. The gene encoding *bla* was amplified from *Yersinia pseudotuberculosis* strain pIB102 *yopE-bla*. The pBAD24 plasmid was cleaved by EcoRI and HindIII (pSB22 and pSB23) or EcoRI and SphI (pRN69, pRN70, pRN71 and pRN72) and each *yopN* variant were introduced into the plasmid together with the *bla* gene using InFusion HD cloning kit as described above. All clones were confirmed by DNA sequencing (GATC Biotech AB, Stockholm, Sweden). The plasmids carrying a C-terminal fusion of *yopN* variants to the *bla* were electroporated into *Yersinia pseudotuberculosis* YPIII/pIB102 (wt strain). The plasmid carrying wt *yopN-bla* fusion was also introduced into a Δ*yopB* mutant (pIB604) [59].

### Generation of in cis yopN mutants

Using PCR, the genes encoding *yopN_RARA_* and *yopN_GAGA_* were amplified from pRN65 and pRN68 and XbaI and SphI sites were introduced at the ends. These fragments were then cloned into the pDM4 suicide plasmid [60] cut with XbaI and SphI resulting in pRN74 (*yopN_RARA_*) and pRN76 (*yopN_GAGA_*). The *E. coli* S17-1 λpir containing either pRN74 or pRN76 were used as donor strains in conjugations with *Yersinia pseudotuberculosis* strains YPIII/pIB102 and Xen4 YPIII/pIB102. After sucrose counter-selection, clones carrying the *yopN*  mutations were isolated. The resulting strains *Y. pseudotuberculosis* YPIII pIB823 (*yopN_RARA_*), pIB825 (*yopN_GAGA_*) and Xen4 pIB825 (*yopN_GAGA_*) were confirmed by PCR and DNA sequence analysis (GATC Biotech AB, Stockholm, Sweden).

YopH_6-99_-Bla or YopE_6-89_-Bla fusions were also introduced into *yopN_GAGA_* and Δ*yopB* mutants using *E.coli* S17-1 λpir containing pNQ705-H-Bla or pNQ705-E-Bla plasmids as donor strains [61]. Both fusions were introduced with single recombination events.

### Analysis of Yop expression and secretion

Overnight cultures of different *Yersinia* strains were grown at 26°C in brain heart infusion (BHI) medium. Next day, they were diluted to the same optical density at 600 nm (OD_600_) in either BHI containing 2.5 mM CaCl_2_ (T3SS non-inducing conditions) or BHI containing 5 mM EGTA and 20 mM MgCl_2_ (T3SS inducing conditions) and grown for 1 h at 26°C. Then cultures were grown at 37°C for additional 3 h after adding arabinose to a final concentration of 0.2% (w/V) when necessary. For analysis of total expression levels, sample buffer (SDS-PAGE) was added directly to whole culture samples. For analysis of secreted proteins, cultures were centrifuged to separate bacteria from supernatant. The supernatants were filtered through 0.45 µm sterile filters and the supernatants were precipitated using 10% trichloroacetic acid (TCA) and centrifuged for 20 minutes. Resulting pellets were resuspended in 0.5 ml 2% SDS. A second round of precipitation was done by mixing the samples with 1.5 ml ice-cold acetone for 30 min at −20°C followed by centrifugation for 20 minutes at 15,000 x g. The final pellets were resuspended with sample buffer. All samples were kept at −20°C prior to analysis.

Proteins were separated by SDS-PAGE. Expression levels were determined by Western blotting and secretion levels were visualized by either Coomassie blue staining or Western blotting.

Primary antibodies used are as follows: anti total-Yop [62] (1/3000) and anti-HA-tag (1/5000; Sigma-Aldrich [now MERCK]). The secondary antibody used was anti-rabbit-horseradish peroxidase (HRP) (1/20,000; Amersham).

### Yop expression and secretion early after Ca^+2^ depletion

Overnight cultures of the different strains grown at 26°C were diluted to OD_600_ 0.1 in BHI containing 2.5 mM CaCl_2_ (non-inducing conditions) and grown for 1 h at 26°C and then grown an additional hour at 37°C. Thereafter, the T3SS was induced by adding MgCl_2_ and EGTA at a final concentration of 20 mM and 10 mM respectively. Growth was continued for an additional 30 min at 37°C. Yop expression levels were analysed on whole cell samples and secretion levels were analysed on filtered supernatants precipitated with TCA as described above.

Proteins were separated by SDS-PAGE. Expression levels were determined by Western blotting.

### Analysis of acid phosphatase from secreted YopH (PTPase assay)

YopH secretion levels were quantified by analysis of acid phosphatase activity. The different strains were grown as described above for analysis of Yop expression/secretion early after Ca^+2^ depletion. Triton X-100 was added to the culture media to a final concentration of 0.1% to promote the release of surface localized YopH.

At the end of the 30 min induction of Yop secretion, OD_660_ values were measured, bacteria were removed by centrifugation and 100 µl of supernatants were analysed directly for PTPase activity.

The PTPase assay was performed as described previously [47,63] with some minor modifications. The assay was performed at 30°C in a total volume of 200 µl containing 20 mM p-Nitrophenyl phosphate (Calbiochem) as substrate, 50 mM citrate pH 5.0 buffer and 2 mM DTT as a reducing agent. After 10 min of incubation, the reaction was stopped by adding 1 ml 0.2 M NaOH and the absorbance at 410 nm was measured. Using the molar extinction coefficient of 1.78 × 10^4^ M^−1^ cm^−1^, the activity of secreted YopH was calculated as 1 unit is defined as 1 mole of p-Nitrophenolate ion produced per (min x ml supernatant x bacterial OD_660_). Statistical analysis of the results was done by Mann-Whitney test.

### Translocation assay

The day before the experiment, 2 × 10^4^ HeLa cells were seeded in triplicates in 96-well plates (PS, F-bottom [chimney well] µClear, black, medium binding; Greiner Bio-One). *Yersinia* strains encoding Yop-Bla fusion proteins were grown in LB with appropriate antibiotics overnight. The next day the *Yersinia* strains were diluted to an OD_600_ of 0.1 in colorless MEM (Gibco) supplemented with 10% FCS and 2 mM glutamine. In addition, CaCl_2_ was added to a final concentration of 2.5 mM (T3SS non-inducing conditions). The different strains were grown at 26°C for 1h and then arabinose was added to a final concentration of 0.2% when necessary. Cultures were then shifted to 37°C and grown for 2 h. HeLa cells were washed three times with supplemented colorless MEM. Where appropriate, arabinose was added to 0.2% into the wells, as well. Infection was initiated by adding bacteria to the wells containing HeLa cells at a multiplicity of infection (MOI) of 25:1 in colorless MEM. After 30 minutes, the infection was stopped by the addition of gentamycin to a final concentration of 25 µg/ml into the wells. 6 x CCF4-AM substrate mix (Live-Blazer fluorescence resonance energy transfer [FRET] B/G substrate kit; Thermo Fischer Scientific) was prepared according to the manufacturer’s protocol with addition of 15 mM probenecid. The substrate mix was added to the wells and incubated for 2 h at room temperature in darkness. Using TECAN Infinite M200 plate reader, green and blue fluorescence was detected according to manufacturer’s protocol. The levels of translocation were calculated as the ratio of blue to green (B:G) signal after subtracting background fluorescence. Values were normalized to a sample indicated in the figure legends. Statistical analysis of the results was done by one-way analysis of variance (ANOVA) followed by the Bonferroni posttest.

### Animal infections

Eight-week old female BALB/c mice were purchased from Taconic and they were given food and water *ad libitum*. The study was approved by the local animal ethics committee (Dnr A54-15).

For oral infection, mice were deprived of food and water 16 h prior to infection. The bacterial suspension of bioluminescent *Yersinia pseudotuberculosis* Xen4 pIB102 (wt) and Xen4 pIB825 (*yopN_GAGA_*) strains were prepared from overnight cultures grown at 26°C into sterile tap water with 150 mM NaCl (4 x 10^8^ CFUs/ml). The mice were allowed to drink bacterial suspensions for 6 h. Then, food and water was provided. Infection doses were calculated according to CFU counts of serially diluted bacterial suspensions together with the measured volumes of contaminated water that was consumed by the mice.

Weights of the mice and signs of disease were followed regularly. At indicated time points or when mice were terminally ill, they were sacrificed.

### Analysis by In Vivo Imaging System (IVIS)

Emitted light from infecting bacteria was monitored and analysed by using a Spectrum In Vivo Imaging System Instrument (Caliper Life Sciences/PerkinElmer). Every second day, mice were anaesthetized with a 25% mixture of Isofluorane (IsoFlo vet: Orion Pharma) in oxygen and thereafter placed in the IVIS under continued isofluorane anaesthesia. Images generated were analysed with the Living Image software, version 4.5.4 (Caliper Life Sciences/PerkinElmer). Bioluminescent signals emitted by the bacteria were determined from defined regions of interest (ROIs) with identical sizes from organs dissected out from the mice and expressed as total Flux (photons per second).
